# Comment on “Isolated perineal plaque as the initial presentation of pemphigus vulgaris”

**DOI:** 10.1016/j.jdcr.2026.01.062

**Published:** 2026-03-04

**Authors:** Manjaree Morgaonkar

**Affiliations:** Department of Dermatology, Dr. D. Y. Patil Medical College, Hospital and Research Center, Pune, Maharashtra, India

**Keywords:** delayed diagnosis, first manifestation, genital dermatoses, isolated perineal erosions, pemphigus vulgaris, social stigma, underreported

*To the Editor:* We read with great interest the case report by Workineh et al,^1^ “Isolated perineal plaque as the initial presentation of pemphigus vulgaris.” The authors should be commended for highlighting a diagnostically challenging presentation. We agree with their emphasis on maintaining a high index of suspicion of pemphigus in cases of isolated perinium erosions.

Although isolated and first involvement of perineum and genitals is rarely reported, mucosal involvement in pemphigus vulgaris (PV) is well recognized. Among all mucosal sites, oral involvement is the most frequently described in scientific literature.^2^ Oral lesions often precede skin involvement or remain the sole site of disease. This pattern reflects the predominant expression of desmoglein-3 in mucosal epithelium.^1^^,^^3^ Oral mucosal lesions are easily recognizable, interfere with feeding, and speech leads to prompt earlier medical attention. This partly explains their over presentation as the commonest and initial manifestation in PV.

Desmoglein-3 is also expressed predominantly in nonoral mucosal sites, including the perineum and genital mucosa. As a result, these areas may be involved early or in isolation.^3^ We suggest that the low incidence of genital or perineal-only PV reflects underreporting rather than true rarity. Genital and perineal lesions are often associated with fear, embarrassment, and notably social stigma.^4^ Their frequent misattribution to sexually transmitted diseases can lead to patient reluctance to report symptoms.^5^

The aforementioned factors can cause delay or underreporting of isolated perineal/genital PV. Early referral to dermatologists, addressing the patient stigma, and high suspicion of isolated PV can increase reporting of this entity.

## Conflicts of interest

None disclosed.
